# Regorafenib in combination with silybin as a novel potential strategy for the treatment of metastatic colorectal cancer

**DOI:** 10.18632/oncotarget.20054

**Published:** 2017-08-07

**Authors:** Valentina Belli, Vincenzo Sforza, Claudia Cardone, Erika Martinelli, Giusi Barra, Nunzia Matrone, Stefania Napolitano, Floriana Morgillo, Concetta Tuccillo, Alessandro Federico, Marcello Dallio, Carmelina Loguercio, Antonietta Gerarda Gravina, Raffaele De Palma, Fortunato Ciardiello, Teresa Troiani

**Affiliations:** ^1^ Oncologia Medica, Dipartimento di Internistica Clinica e Sperimentale “F. Magrassi”, Università degli Studi della Campania “Luigi Vanvitelli”, Napoli, Italy; ^2^ Medicina Interna, Dipartimento di Internistica Clinica e Sperimentale “F. Magrassi”, Università degli Studi della Campania “Luigi Vanvitelli”, Napoli, Italy; ^3^ Gastroenterologia, Dipartimento di Internistica Clinica e Sperimentale “F. Magrassi”, Università degli Studi della Campania “Luigi Vanvitelli”, Napoli, Italy

**Keywords:** metastatic colorectal cancer (mCRC), regorafenib, silybin, reactive oxygen species (ROS)

## Abstract

**Purpose:**

Regorafenib, an oral multikinase inhibitor, has demonstrated survival benefit in metastatic colorectal cancer (mCRC) patients that have progressed after all standard therapies. However, novel strategies to improve tolerability and enhance anti-cancer efficacy are needed.

**Experimental design:**

We have evaluated *in vitro* the effects of regorafenib in combination with silybin, a biologically active component extracted from the seeds of Silybum marianum, in a panel of human colon cancer cells. Furthermore, we have prospectively treated a cohort of 22 refractory mCRC patients with regorafenib plus silybin.

**Results:**

Treatment with regorafenib determined a dose-dependent growth inhibition whereas treatment with silybin had no anti-proliferative effects among all cancer cells tested. The combined treatment with regorafenib and silybin induced synergistic anti-proliferative and apoptotic effects by blocking PI3K/AKT/mTOR intracellular pathway. Moreover, combined treatment with regorafenib and silybin increased the production of reactive oxygen species levels within cells. In an exploratory proof of concept clinical study in a cohort of 22 mCRC patients after failure of all standard therapies, the clinical activity of regorafenib in combination with silybin was assessed. A median progression-free survival of 10.0 months and a median overall survival of 17.6 months were observed in these patients. These results suggest that the combined treatment potentially increases the clinical efficacy of regorafenib. Moreover, due to its anti-oxidative properties, silybin could protect patients from drug-induced liver damages, allowing to continue an effective anti-cancer therapy.

**Conclusions:**

The present study suggests that silybin in combination with regorafenib is a promising strategy for treatment of metastatic colorectal patients.

## INTRODUCTION

Novel advances in metastatic colorectal cancer (mCRC) therapies have led over the last two decades to an increase in median overall survival (OS) of patients from 12 months, during the 5-fluoruracil (5-FU)-based chemotherapy era to approximately 30 months due to development of new therapeutic agents [1–2]. In this scenario, regorafenib is an oral multikinase inhibitor that targets different protein kinases involved in key oncogenic pathways, such as angiogenesis by blocking vascular endothelial growth factor receptor-1 (VEGFR-1), 2 and 3 and tyrosine kinases, immunoglobulin and epidermal growth factor (EGF) homology domain 2 (TIE-2) tyrosine kinase; such as tumor progression by inhibition KIT, RET, RAF-1 and BRAF; and such as tumor microenvironment by blocking platelet derived growth factor receptor-β (PDGR-β) and fibroblast growth factor receptor (FGFR) tyrosine kinases [3–4]. Two phases III clinical trials (CORRECT and CONCUR) have demonstrated in heavily pre-treated mCRC patients a significant improvement in OS and in progression free survival (PFS) with regorafenib as compared to placebo [5–6]. Despite the advantage in survival, very few objective responses have been reported with the main effect consisting in disease stabilization [5–7]. Based on these results, regorafenib has been approved by the European Medicines Agency (EMA) and the Food and Drug Administration (FDA) for the treatment of chemorefractory mCRC patients. However, the toxicity profile, including liver toxicity, fatigue and hand and foot skin reactions (HFSR), has limited drug use in clinical practice [8]. These concerns have produced an urgent need for the identification of compounds that are able to limit regorafenib side effects and, therefore, for improving regorafenib tolerability and, at the same time, enhancing its clinical activity. In this scenario, silybin is a biological active compound that is extracted from the milk thistle seeds of *Silybum marianum*, which has been shown to have anti-inflammatory and anti-oxidative effects in the treatment of liver diseases, such hepatitis and cirrhosis [9–10]. Moreover, in the last two decades, silybin has shown anti-cancer effects against several types of tumor models, mainly through targeting cancer cell proliferation, metabolism, apoptosis, inflammation, or angiogenesis [11–19]. Silybin has been conjugated with vitamin E and phospholipids to improve the bioavailability, that is lower when pure silybin is administered orally, and, therefore, its antioxidant activity (RealSiL). In this formulation, it has been proposed as a potential agent for reducing drug-induced liver damage [20–22]. Based on these considerations, we have performed an *in vitro* study to evaluate the ability of silybin in combination with regorafenib to inhibit the growth and induce apoptosis in a panel of human colon cancer cell lines. Furthermore, we have explored the potential clinical role of combined treatment in a prospective cohort of 22 mCRC patients that have progressed following administration of all available standard therapies.

## RESULTS

### Sensitivity to regorafenib and silybin treatment in a panel of human colon cancer cell lines

**Figure 1:**
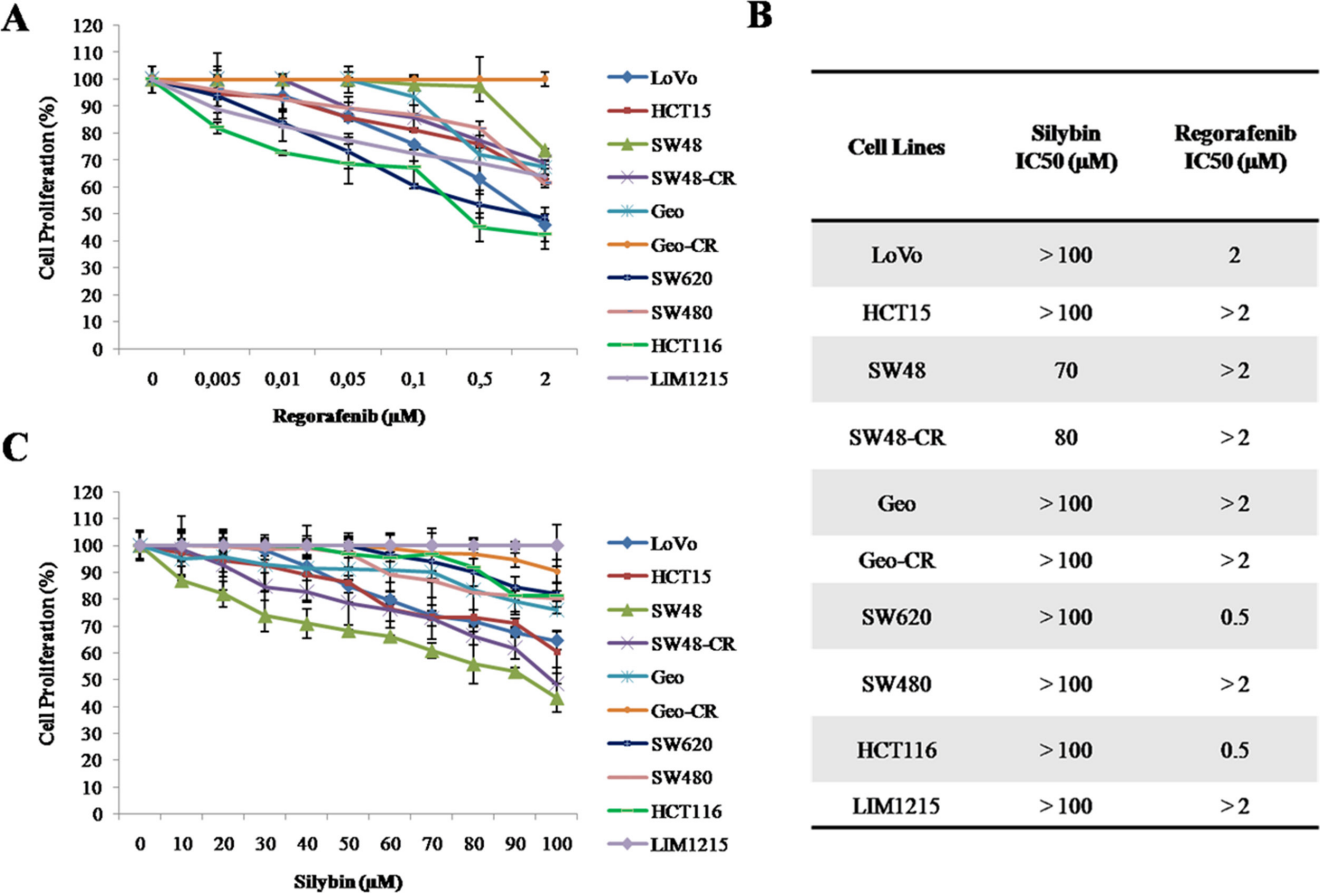
Effects of regorafenib or silybin treatment on cell proliferation in a panel of human colon cancer cell lines (**A–C**) Cells were treated with different concentrations of regorafenib (range, 0.005–2 μM) or silybin (range, 10–100 μM) for 96 hours. The proliferation rate was evaluated by MTT assay, as described in Materials and Methods. (B) The IC_50_ value was determined by interpolation with dose-response curves. Results represent the mean of three separate experiments, each performed in duplicate.

To evaluate the anti-proliferative effects of regorafenib and silybin treatment, as single agents, we have selected ten human colon cancer cell lines (LoVo, HCT15, SW48, SW48-CR, GEO, GEO-CR, SW620, SW480, HCT116 and LIM1215) that have distinct mutation profiles in *KRAS*, *NRAS, BRAF*, and *PIK3CA* genes. In particular, SW48 and LIM1215 cancer cells, that are wild type (WT) for *KRAS, BRAF, NRAS* and *PIK3CA* genes, and GEO cancer cells, that have a *KRAS* codon 12 mutation, are sensitive to the anti-epidermal growth factor receptor (EGFR) monoclonal antibody cetuximab [23]. Despite GEO cells harbors a *KRAS* gene mutation, previous studies from different laboratories including our own, have demonstrated that this cancer cell line is one of the most sensitive to *in vitro* and *in vivo* anti-tumor activity of cetuximab treatment [24]. Moreover, we have selected two cetuximab-acquired resistant cell lines, such as GEO-CR and SW48-CR, that have been previously obtained in our laboratory [25], and five cetuximab-primary resistant cell lines, such as LoVo, HCT15, SW620, SW480 and HCT116, that have an activating mutation in the *KRAS* gene in either codon 12 or 13 within exon 2. All cancer cells were exposed to escalating doses of regorafenib (range, 0.005–2 μM) or silybin (range, 10–100 μM), for 96 hours and the effects on cell proliferation were evaluated by 3-(4,5-dimethylthiazol-2-yl)-2,5-diphenyltetrazolium bromide (MTT) assay. The drug concentrations required to inhibit cell growth by 50% (IC_50_) were determined by interpolation from the dose-response curves. As shown in Figure 1A–1B, there was a differential sensitivity to regorafenib-induced cell growth inhibition. In fact, three colon cancer cell lines (HCT116, SW620 and LoVo) were the most sensitive to regorafenib treatment, with IC_50_ values ranging from 0.5 μM (HCT116 and SW620) to 2 μM (LoVo). Silybin had no anti-proliferative effects among the colon cell lines harboring *KRAS, NRAS, BRAF*, or *PIK3CA* mutations (Figure 1B–1C).

### Effects of regorafenib in combination with silybin on cell proliferation in a panel of human colon cancer cell lines

We next evaluated the anti-proliferative activity of regorafenib in combination with silybin in the panel of human colon cancer cell lines. In particular, cancer cells were treated with two different concentrations of silybin, 60 and 90 μM, with increasing doses of regorafenib (range, 0.005–2 μM) for 96 hours. In all cancer cell lines tested, except for the cetuximab-sensitive LIM1215 cells, combined treatments determined a significant anti-proliferative effect in a dose-dependent manner as compared to single treatments (Supplementary Figure 1). In particular, while regorafenib treatment had no effect on cell growth in HCT115 cells, the combined treatment with regorafenib 0.01 μM and silybin 90 μM restored the sensitivity of HCT115 cells to regorafenib (Supplementary Figure 1). Moreover, no significant differences were observed between 60 and 90 μM of silybin.

### Effects of regorafenib treatment in combination with silybin on colony formation of SW48, SW48-CR, HCT15 and SW480 colon cell lines

**Figure 2:**
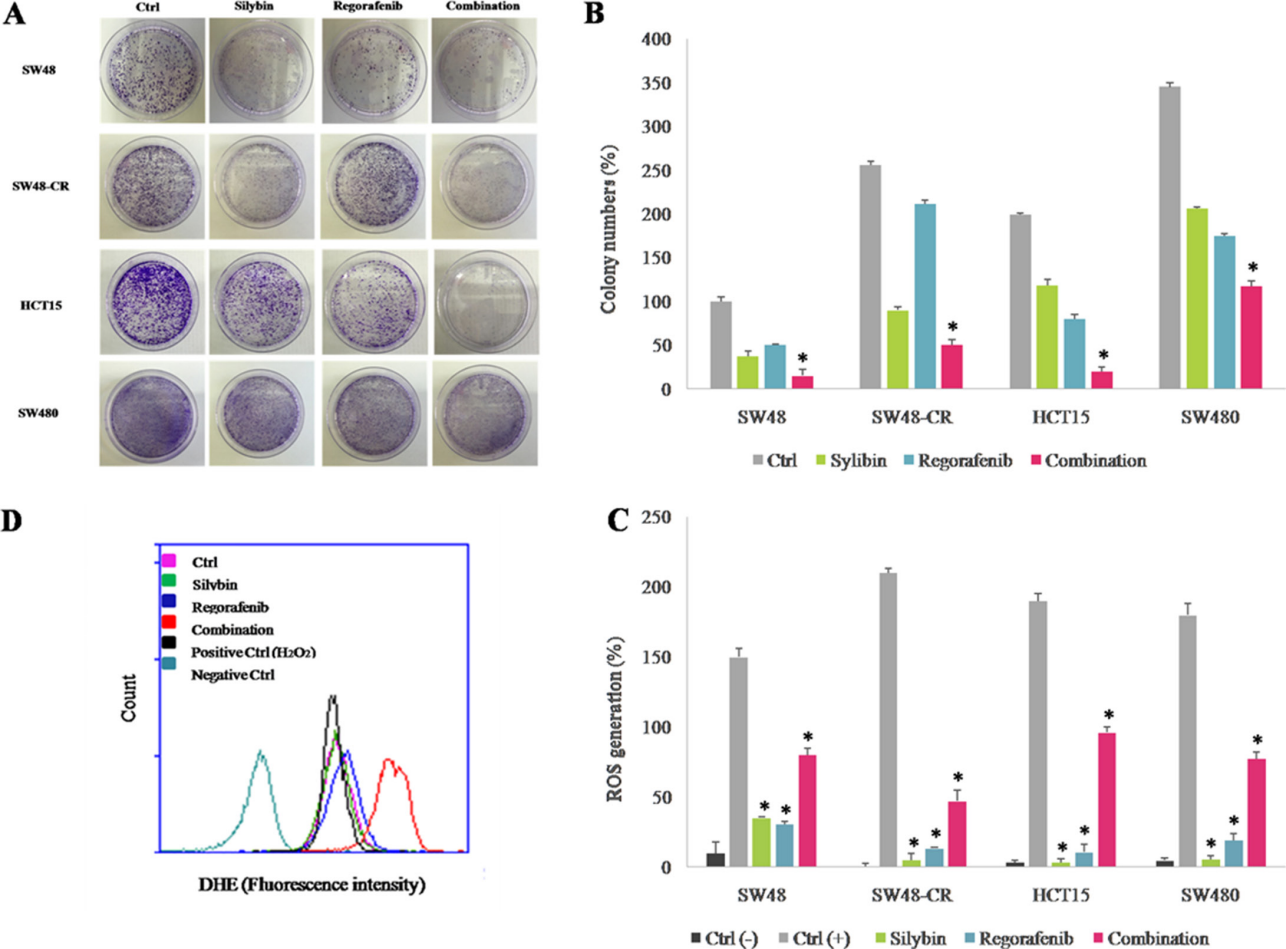
Effect of regorafenib in combination with silybin on colony formation and on cell metabolism in SW48, SW48-CR, HCT15 and SW480 colon cancer cells lines The SW48, SW48-CR, HCT15 and SW480 cancer cells were treated with silybin (90 μM) or regorafenib (2 μM) and their combination for 72 hours. After 14 days the colonies were stained with 0.1% crystal violet and counted as described in Materials and Methods. (**A**) Images of colon cancer cells acquired by phase-contrast microscope. (**B**) Histogram of colony number counted by image j plugin. Error bars indicate the standard deviation. *p < 0.05 compared to single treatment. The effects of treatments were also evaluated in terms of oxidative stress generation as described in Materials and Methods. (**C**) Histogram of DHE mean fluorescence intensity (% of control). Error bars indicate the standard deviation. *p < 0.05 compared to positive control. (**D**) Flow cytometry overlay of dihydroethidium (DHE) fluorescence intensity within HCT15 cells.

To further evaluating the anti-cancer activity of combined treatment, we performed a colony formation assay. To do this, we selected four cell lines with different mutation profile for KRAS gene (SW48, SW48-CR, HCT15 and SW480 cells) and in which the treatment induced a stronger anti-proliferative effect compared to all cells tested. Hence, cancer cells were treated with silybin (90 μM) and regorafenib (2 μM) as single agents or in combination for 72 hours. After 14 days colonies were stained with crystal violet and counted (Figure 2A–2B). In the control group, cancer cells formed large cell clusters within 48 hours. On the contrary, combination treatment reduced significantly the number of colonies compared to control and to single agent in all cancer cell lines.

### Effects of regorafenib treatment in combination with silybin on cell metabolism in SW48, SW48-CR, HCT15 and SW480 colon cell lines

We investigated the effect of combined treatment on cell metabolism by evaluating the reactive oxygen species (ROS) production in cancer cells. Oxidative stress occurs in cells when the generation of ROS overwhelms the cell natural antioxidant defenses. Several studies have demonstrated that several anti-cancer drugs could lead to ROS production and that this effect could play an important role in their anti-cancer activity [26–27]. The ROS accumulation was evaluated by dihydroethidium (DHE) assay using flow cytometry in SW48, SW48-CR, HCT15 and SW480 cells. As shown in Figure 2C–2D, ROS levels significantly increased following combination treatment in all cells; whereas single treatments had no effect on intracellular ROS generations. These data suggest that combined treatment of regorafenib plus silybin increases the production of ROS and accumulation of superoxide anions.

### Effect of regorafenib treatment in combination with silybin on apoptosis induction in SW48, SW48-CR, HCT15 and SW480 cells

**Figure 3:**
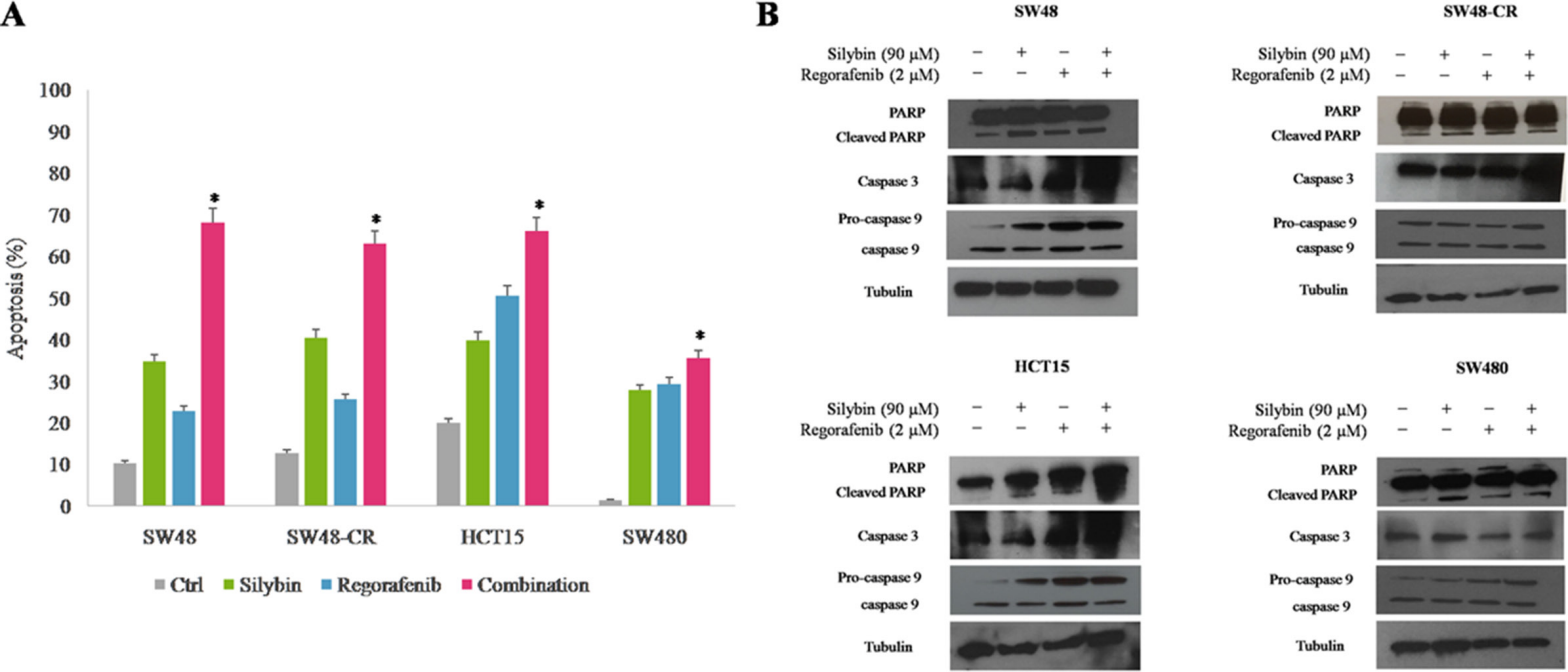
Effects of regorafenib in combination with silybin on induction of apoptosis in SW48, SW48-CR, HCT15 and SW480 colon cancer cells (**A**) Apoptosis was evaluated with Annexin-V-FITC staining and 7-Amino-Actinomycin D (7-AAD) detection assays using flow cytometry in SW48, SW48-CR, HCT15 and SW480 cancer cells after 24 hours of incubation with silybin (90 μM) or regorafenib (2 μM) and their combination. Histogram of data expressed as percentage of apoptotic cells.*p < 0.05 compared to single treatment. (**B**) Colon cancer cells were treated with silybin (90 μM) or regorafenib (2 μM) and their combination for 24 hours. Expression of PARP, caspase 3 and 9 were evaluated by immunoblotting as described in Materials and Methods. α-Tubulin was used as the loading control.

Furthermore, we measured the ability of regorafenib and silybin in combination to induce apoptosis by using Annexin V-FITC assay. As depicted in Figure 3A, compared to single agents, the combined treatment induced significantly more early and late apoptosis within 24 hours of treatment in all colon cells. In particular, SW48 cells showed an apoptotic rate of 22% and 34%,with regorafenib or silybin treatment, respectively; whereas the combined treatment induced an apoptotic rate of 62%. To further expand these results, the activation status of different pro-apoptotic factors was examined by western blotting. The combined treatment with regorafenib and silybin substantially induced the cleavage of PARP fragment in all cell lines (Figure 3B). Moreover, we observed a strong increasing in expression levels of caspase 3 and pro-caspase 9 in SW48, SW48-CR and HCT15 cells, suggesting a probable activation of extrinsic apoptotic mechanisms involving caspase 9- independent and caspase 3-dependent pathway in these cell lines [28]. Conversely, the expression levels of both caspases were approximately the same in SW480 cells and this effect could be explained by the activation of caspase 9- and caspase 3- independent mechanisms. Further experiments are needed to clarify the mechanisms involved in apoptosis process after regorafenib and silybin treatment.

### Effects of regorafenib treatment in combination with silybin on PI3K/AKT/mTOR intracellular pathway in SW48, SW48-CR, HCT15 and SW480 cells

**Figure 4:**
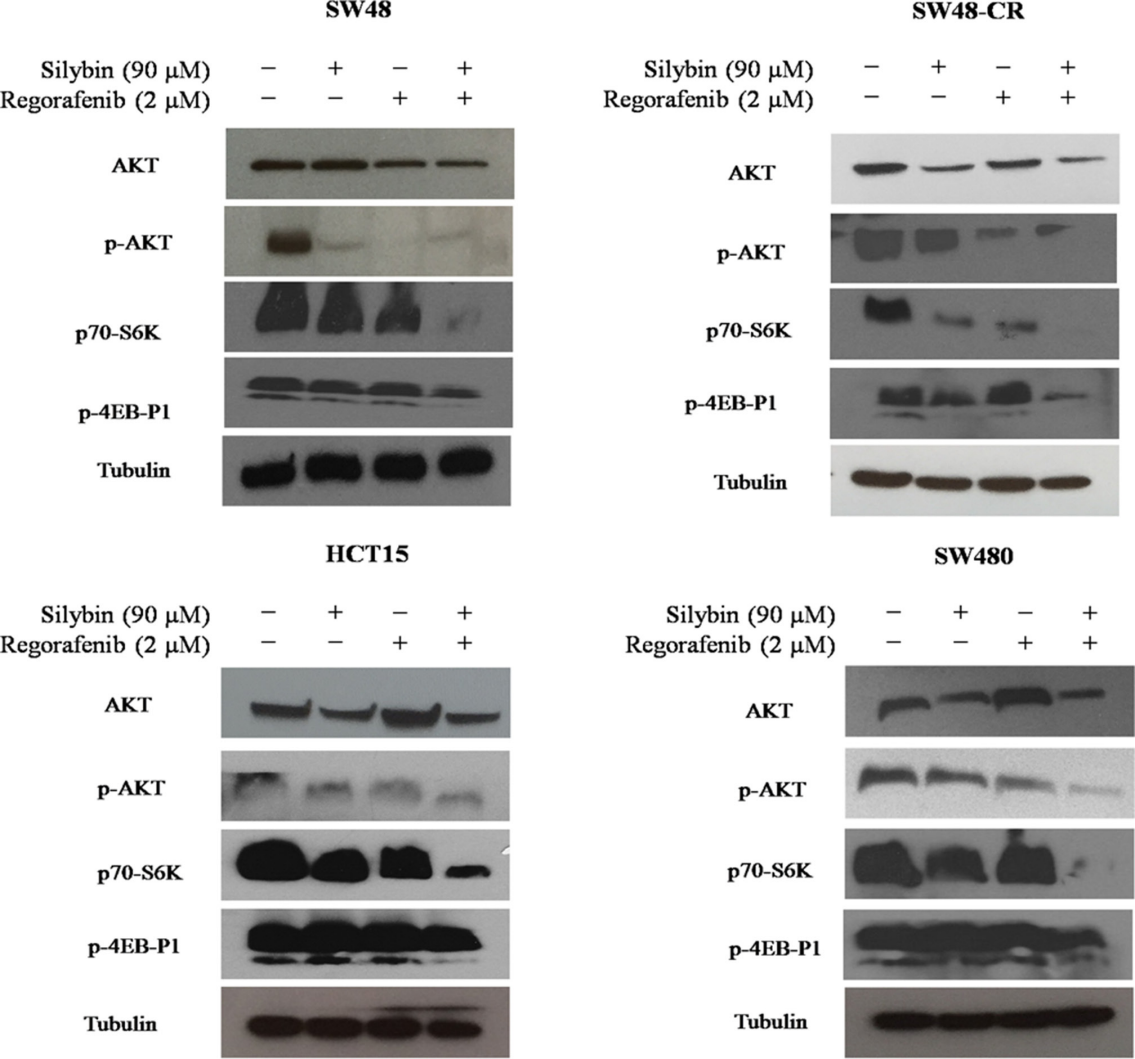
Effects of regorafenib in combination with silybin on PI3K/AKT/mTOR intracellular signaling in SW48, SW48-CR, HCT15 and SW480 colon cancer cell lines SW48, SW48-CR, HCT15 and SW480 cancer cells were treated with silybin or regorafenib and their combination at the indicated doses for 24 hours. Total cell protein extracts were subjected to immunoblotting with the indicated antibodies as described in Materials and Methods. α-Tubulin was used as the loading control.

To examine the mechanisms by which the combined treatment inhibit cancer cell proliferation, the activation of the PI3K/AKT/mTOR pathway was evaluated. The SW48, SW48-CR, HCT15 and SW480 cells were treated with silybin (90 mM) or regorafenib (2 mM) or with their combination for 24 hours. The activation of PI3K/AKT/mTOR signaling was analyzed by western blot analysis. As shown in Figure 4, the combined treatment induced a significant inhibition of the expression of the total form of the AKT protein as compared to single treatment. Interestingly, the combination of regorafenib plus silybin substantially inhibited the phosphorylation of AKT (pAKT) in all cancer cells. In addition, we studied also the expression of ribosomal S6 kinase (p70-S6K) and 4EB-P1 protein (p4EB-P1), two major effectors of PI3K/AKT/mTOR signaling. We observed a greater inhibition of p70-S6K and p4EB-P1 after combination treatment compared to regorafenib or silybin alone. This effect was more evident in SW48, HCT15 and SW480 cells (Figure 4).

### Clinical activity and tolerability of regorafenib in combination with silybin in metastatic colorectal cancer patients

**Figure 5:**
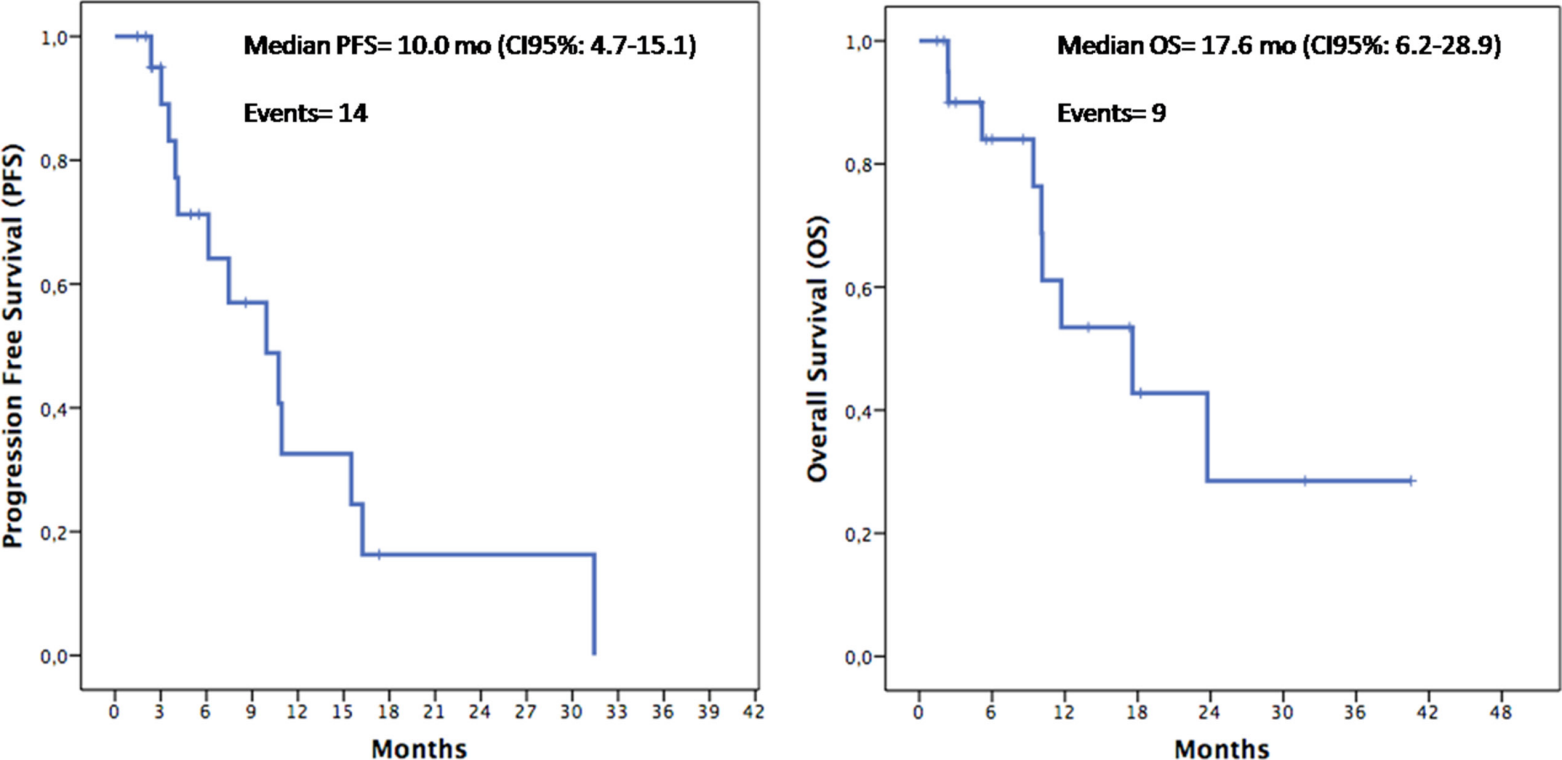
Kaplan–Meier analysis of Progression-Free Survival (PFS) and Overall Survival (OS) in the overall population

**Figure 6:**
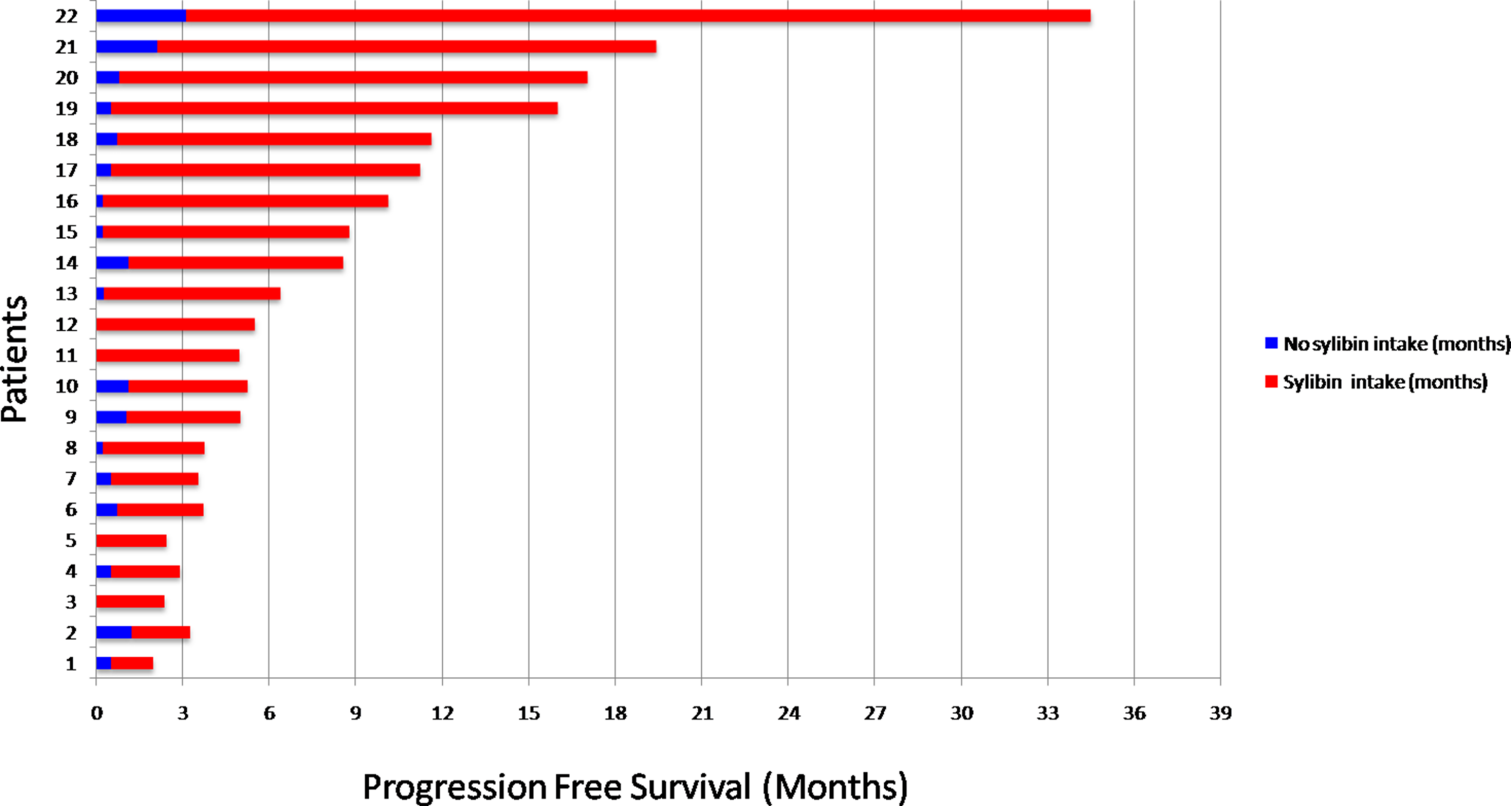
Progression Free Survival (PFS) in the overall population Patients 1-2-5-6-11-12-15-21: treatment was ongoing at the time of data cutoff; Patients 3-5-11-12 received silybin treatment from the first cycle due to basal hypertransaminasemia.

**Figure 7:**
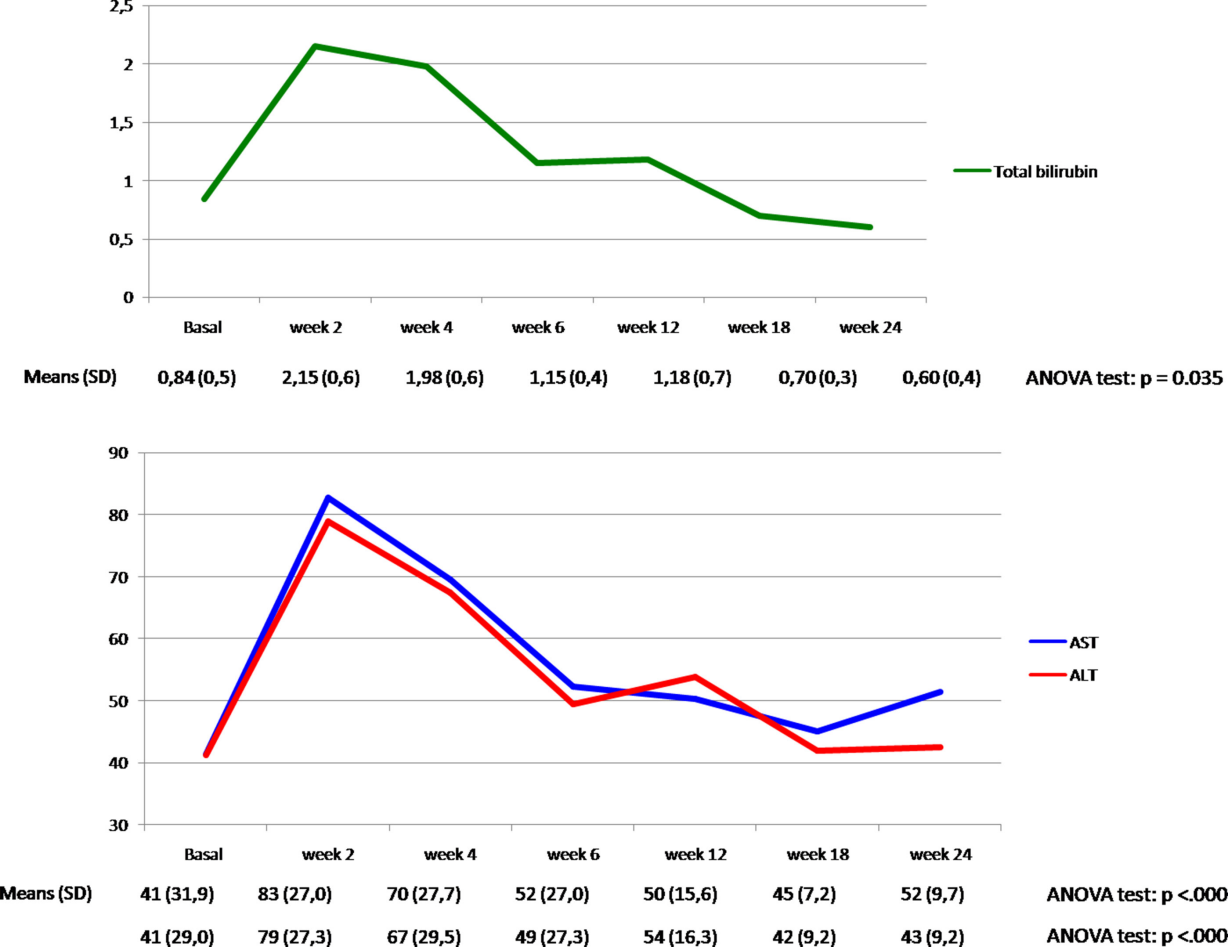
Total bilirubin, AST and ALT plasma levels (mean ±) in the overall population during treatment with regorafenib and silybin

To evaluate if silybin could increase the clinical efficacy and the toxicity profile of regorafenib, a pilot single arm clinical study was conducted. From November 2013 to March 2017 twenty-two mCRC patients after failure of all available treatments including anti-angiogenic drugs and anti-EGFR monoclonal antibodies (in RAS wild type disease) were treated with regorafenib. Silybin-vitamin E-phospholipids complex (188 mg of silybin, 388 mg of phosphatidylcholine, and 60 mg of vitamin E/day) was added to regorafenib treatment at the occurrence of liver toxicity. Baseline demographics, clinical and pathologic characteristics are listed in Supplementary Table 1. In this respect, 17 out of 22 patients (77%) had a history of metastatic disease longer than 18 months and in 17 patients (77%) the number of metastatic sites were ≥ 2. Eleven out of 22 patients (50%) received ≥ 3 therapies for treatment of metastatic disease before regorafenib. The performance status (PS) according to ECOG scale was 0 in 14 patients (64%) (Supplementary Table 1). In these patients, a median PFS of 10.0 months (mo) (CI95%: 4.7–15.1) [CI 95%: 4.7–15.1 mo] and a median OS of 17.6 mo [CI 95%; 6.2–28.9 mo] were observed, respectively (Figure 5). Four out of 22 patients (18%) started silybin concomitantly with the first dose of regorafenib. Two of these patients started with one dose level reduction of regorafenib (120 mg) because of baseline liver impairment (G2 blood bilirubin increase and G1 increase in blood levels of AST and ALT) and did not require any further regorafenib dose reduction during treatment. The other two patients started with a dose of 80 mg of regorafenib (two dose level reductions) because of concomitant PS 2, according to ECOG scale, G2 hyperbilirubinemia andG1 hypertransaminasemia (Supplementary Table 2). Three of these 4 patients are, as of May 16, 2016, receiving the combined treatment with regorafenib and silybin. Eighteen out of 22 patients (82%) started treatment with silybin at the occurrence of liver toxicity, mainly during the second week of treatment and this was continued until disease progression (Figures 6 and 7). Eighteen out of 22 patients (82%) required a reduction of regorafenib dose during treatment. In particular, sixteen patients (72%) required a dose reduction of regorafenib during the first three cycles, mainly for hyperbilirubinemia and/or hypertransaminasemia (85%) or for hand and foot skin reactions (HFSR) (15%), whereas in 2 patients (9%) the dose was reduced after the third cycle of treatment for HSFR and fatigue. Six patients out of 22 (27%) needed one dose level reduction of regorafenib (120 mg), while 12 patients (55%) required 2 dose levels reduction (80mg) (Supplementary Table 2). When the median values of transaminase and bilirubin blood levels in patients treated with regorafenib were evaluated, an increase from baseline to the second week of the first cycle of treatment with regorafenib was observed, whereas a rapid reduction in transaminase and bilirubin blood levels was found once treatment with silybin was started (Figure 7). The effect of silybin on restoring hepatic function was significant for both the normalization of AST and ALT levels (*p* < .0001) and for the reduction of bilirubin levels (*p* = 0.035). No unexpected toxicities were observed with the combined treatment with regorafenib and silybin. The reason of discontinuation from regorafenib treatment was radiological progression of disease in 14 out of 22 patients (64%), while eight patients (36%) are still on treatment. We did not observe any discontinuation of treatment due to adverse events.

## DISCUSSION

The development of molecularly targeted therapies has provided novel opportunities for improving and personalizing the treatment of mCRC. In this scenario, the multikinase inhibitor regorafenib was demonstrated, as compared to placebo, to significantly increase median OS in two large phase III randomized trials in chemorefractory mCRC patients. However, the toxicity profile, in particular liver toxicity, as well as the lack of predictive biomarkers of response, limits its use in clinical practice.

Several experimental studies have provided evidence for anti-inflammatory and anti-oxidative effects of silybin, an active constituent of the milk thistle seeds of *Silybum marianum*, for the potential treatment of liver diseases, including protection of liver function damage by drugs, such as cytotoxic anti-cancer agents [9–10]. Moreover, it has been suggested a direct silybin cell growth inhibitory activity on several tumor types, including prostate, skin, bladder and lung cancers [11–15]. In the present study, we have evaluated the potential role of regorafenib and silybin combination as a novel strategy for the treatment of mCRC patients. Here we provide evidence for a synergic anti-proliferative effect of combined treatment in a panel of human colon cancer cell lines. In fact, the treatment of regorafenib plus silybin induced a stronger inhibition of cell proliferation compared to regorafenib and silybin alone. Furthermore, we demonstrated an increasing in ROS production and apoptotic rate after combined treatment in several colon cancer models.

With a therapeutic perspective, we have performed an exploratory proof of concept clinical study, in which we have assessed the clinical activity of regorafenib plus silybin in a consecutive cohort of 22mCRC patients, that were eligible for regorafenib treatment after failure of all available standard anti-cancer therapies. A median PFS of 10.0 mo [CI 95%: 4.7–15.1 mo] and a median OS of 17.6 mo [CI 95%; 6.2–28.9 mo] were observed in these patients. Although these results derive from a small size, single arm, proof of concept, prospective clinical study and although they need to be confirmed and validated in appropriate larger and randomized studies, they suggest that silybin addition to regorafenib treatment in mCRC patients that have failed all available anti-cancer treatments could potentially increase the anti-tumor efficacy of regorafenib. Furthermore, another potentially relevant clinical implication of this study is the evidence that addition of silybin to regorafenib treatment at the occurrence of regorafenib-induced liver toxicity, was associated with a statistically significant decreasing in the serum level of AST, ALT and bilirubin. Therefore, silybin could protect the patient from liver damage induced by regorafenib and, therefore, could allow to continue a potentially effective anti-cancer therapy.

## MATERIALS AND METHODS

### Drugs

Silybin was provided by Indena (Milan, Italy). Regorafenib was kindly provided by Bayer Pharma (Berlin, Germany). For *in vitro* studies, silybin and regorafenib were dissolved in sterile dimethyl sulfoxide (DMSO) and the stock solution (10 mmol/L) was stored in aliquots at −20°C. The working solution was diluted in culture medium just before each experiment.

### Cell lines

The human SW48 colon cancer cell (*KRAS, NRAS, BRAF* and *PIK3CA* wild-type profile) was obtained from IRCCS “Azienda Ospedaliera Universitaria San Martino-IST Istituto Nazionale per la Ricerca sul Cancro, Genova” Italy. The human GEO colon cancer cell line (*KRAS* mutation (G12A); *NRAS, BRAF*, and *PIK3CA* WT was kindly provided by Dr. N. Normanno (National Cancer Institute, Naples, Italy). The human LIM1215 colon cells (*KRAS, NRAS, BRAF* and *PIK3CA* WT) was obtained from Dr.ssa Di Nicolantonio at Candiolo National Cancer Institute (Candiolo, Italy). The human HCT15, HCT116, SW480, LoVo and SW620 colon cancer cell lines, having different mutation profiles in *KRAS*, *NRAS*, *BRAF*, and *PIK3CA* genes, were purchased from ATCC. The GEO-CR and SW48-CR, were established as previously described [25]. The HCT15, SW480, SW48, SW48-CR, LoVo, LIM1215 and HCT116 cancer cells were cultured with RPMI-1640 medium (Sigma-Aldrich, Missouri, USA) supplemented with 10% fetal bovine serum (FBS, Sigma-Aldrich), 100 U/ml penicillin and 100 mg/ml streptomycin. The SW620, GEO and GEO-CR cancer cells were routine culturing with McCoy's medium (Sigma-Aldrich) supplemented with 10% FBS, 100 U/ml penicillin and 100 mg/ml streptomycin. The cells were maintained in a humidified controlled atmosphere with 95% to 5% ratio of air/CO_2_, at 37°C. The medium was changed every 3–4 days.

### Cell viability assay

Colon cancer cells were seeded into 24-well plates at the density of 1 × 10^4^ cells/well and exposed to increasing concentrations of silybin (range: 10–100 μM) or regorafenib (range: 0.005–2 μM). For the combination treatments, colon cancer cells were treated with two concentrations of silybin (60 or 90 μM) with increasing doses of regorafenib (0.005 to 2 μM). The growth inhibition was assessed by 3-(4,5-dimethylthiazol-2-yl)-2,5-diphenyltetrazoliumbromide (MTT, Sigma-Aldrich) assay after 96 h of incubation. The concentrations inhibiting 50% of cell growth (IC_50_) were obtained and these values were used for subsequent experiments. Results represent the median of three separate experiments, each performed in duplicate.

### Colony formation assay

Colony formation test was performed to evaluate the long-term proliferative potential of SW48, SW48-CR, HCT15 and SW480 cells following silybin and regorafenib treatments. Cancer cells were seeded in 100 mm^3^ cell culture dishes at the density of 3 × 10^3^ and incubated with silybin (90 μM) or regorafenib (2 μM) and their combination for 72 hours. The medium was replaced with fresh culture media every 3 days. After 14 days, cells were fixed with 4% paraformaldehyde at room temperature (RT) for 15 minutes, stained with 0.1% crystal violet and counted using image j plugin [29]. Results represent the median of two separate experiments.

### Reactive oxygen species (ROS) detection

The evaluation of ROS accumulation was detected using dihydroethidium (DHE) assay by flow cytometry (BD Accuri™ C6 Plus) [30]. Once oxidezed within cells, DHE was converted into ethidium (HE) and emits at 605 nm. Briefly, the SW48, SW48-CR, HCT15 and SW480 cancer cells were seeded in 6-well plates (1× 10^5^ cells/well) and treated with silybin (90 μM) or regorafenib (2 μM) and their combination for 24 hours. Cancer cells were also treated with 250 μM of H_2_O_2_ as positive control of cytotoxicity. As a negative control, we applied the same assay onto untreated cells. At the end of each treatment, all cancer cells were incubated with 20 ng/mL DHE stock solutions (2.5 mg/mL) for 1 hours. At the time of processing, cells were harvested, washed twice with PBS 1X and the pellet was suspended in 500 μl of PBS 1X. The dye accumulation was measured using BD Accuri™ C6 (BD Bioscences) flow cytometer and analyzed by BD Accuri™ C6software. For each sample, 1 × 10^4^ events were acquired. Results represent the median of three separate experiments, each performed in duplicate.

### Apoptosis assay

Apoptotic cell death was analyzed using Annexin-V-FITC and 7-Amino-Actinomycin D (7-AAD) detection assays (Invitrogen). Briefly, SW48, SW48-CR, HCT15 and SW480 were seeded in 6-well plates at the density of 2 × 10^5^ cells/well with the same concentrations used for ROS evaluation. After 24 h of treatment, cells were harvested, centrifuged for 5 minutes at 1200 RPM and the pellets were suspended in 100 μL 1X Annexin buffer solution. Then, 5 μL Annexin V-FITC and 10 μL 7-AAD (100 μg/ml) were added to cell suspension at RT for 15 minutes. The detection of viable cells, early and late apoptosis cells, and necrotic cells were performed by BD Accuri™ C6 (BD Bioscences) flow cytometer following the manufacturer's protocol. For each sample, 1 × 10^4^events were acquired. Results represent the median of three separate experiments, each performed in duplicate.

### Immunoblotting

The SW48, SW48-CR, HCT15 and SW480 cancer cells were seeded into 100 mm^3^ petri dishes and treated for 24 h with silybin or regorafenib and their combination, as previously indicated. Cells were lysed with RIPA lysis buffer (Sigma-Aldrich, MO, USA) with protease and phosphatase inhibitors cocktail. Protein extracts were then quantified by using Bradford assay (BioRad, CA, USA), according to the manufacturer's instruction. Equal amounts of total proteins were separated by 4–15% gradient mini precast TGX gel (BioRad) and transferred to nitrocellulose membrane (BioRad). The membrane was blocked with 5% of milk at RT for 1 hours and incubated with following primary polyclonal antibodies Caspase 3 (#9662), PARP (#9542), AKT (#9272), p70-S6K (#9205) and monoclonal antibodies Caspase 9 (#9508), p-AKT (#4060) and p4EB-P1 (#2855) purchased from Cell Signaling (Beverly, MA, USA). Monoclonal anti-α-tubulin antibody was provided by Sigma-Aldrich (St. Louis, MO, USA). After incubation with secondary anti-goat antibody at room temperature for 1 hours, according to the manufacturer's instruction, the membranes were developed using an enhanced chemiluminescence (ECL) detection system (Invitrogen, CA, USA). Each experiment was done in duplicate.

### Clinical study: patient population

We performed a single institution, exploratory proof of concept, single arm clinical study to assess the clinical activity of regorafenib in combination with silybin in mCRC patients, after failure of standard therapies including fluoropyrimidine, oxaliplatin, irinotecan, anti-VEGF therapy and anti-EGFR drugs if *RAS* WT. Regorafenib was administrated at a dose of 160 mg/day for the first 3 weeks q28, whereas silybin-vitamin E-phospholipids complex (188 mg of silybin, 388 mg of phosphatidylcholine, and 60 mg of vitamin E/day) was administered continuously, starting at the appearance of liver toxicity, defined as grade 1 (G1) increased level of blood bilirubin and/or G1 increased levels of aspartate aminotransferase (AST) or alanine aminotransferase (ALT), according to National Cancer Institute Common Terminology Criteria for Adverse Events (CTCAE), version 4.03. The study population consisted of a consecutive cohort of 22 patients, older than 18 years with histological confirmed adenocarcinoma of the colon or rectum, Eastern Cooperative Oncology Group (ECOG) performance status (PS) of 0–2. Data were collected from patients who received at least one regorafenib and one silybin dosefrom November 2013 to a data cut-off of the 15th of March 2017. Baseline demographic and clinical characteristics are listed in Supplementary Table 1. All patients provided informed consent before receiving the first dose of regorafenib. Severity of adverse events (AEs) was graded using the CTCAE, version 4. We performed a weekly clinical visit during the first month with a physical and biochemistry assessment. Tumor response was evaluated every 8 weeks and assessed according to the Response Evaluation Criteria in Solid Tumors (RECIST 1.1). OS, PFS and safety were evaluated.

### Statistical analysis

Statistical analyses of the *in vitro* data were performed using a one-way analysis of variance (ANOVA). Quantitative data were reported as mean ± standard deviation (SD). Results were compared by analysis of variance (ANOVA), and a *p* value < 0.05 was considered statistically significant. The Kaplan-Meier method was used to estimate median PFS and OS time of patient population, *p* values were calculated using log-rank tests at a significance level of 5%. ANOVA test was used to detect mean differences on continuous variables. All statistical analyses were performed using IBM-SPSS statistics version 22.0.

## SUPPLEMENTARY MATERIALS FIGURE AND TABLES


