# Correction: Regorafenib in combination with silybin as a novel potential strategy for the treatment of metastatic colorectal cancer

**DOI:** 10.18632/oncotarget.28765

**Published:** 2025-08-29

**Authors:** Valentina Belli, Vincenzo Sforza, Claudia Cardone, Erika Martinelli, Giusi Barra, Nunzia Matrone, Stefania Napolitano, Floriana Morgillo, Concetta Tuccillo, Alessandro Federico, Marcello Dallio, Carmelina Loguercio, Antonietta Gerarda Gravina, Raffaele De Palma, Fortunato Ciardiello, Teresa Troiani

**Affiliations:** ^1^Oncologia Medica, Dipartimento di Internistica Clinica e Sperimentale “F. Magrassi”, Università degli Studi della Campania “Luigi Vanvitelli”, Napoli, Italy; ^2^Medicina Interna, Dipartimento di Internistica Clinica e Sperimentale “F. Magrassi”, Università degli Studi della Campania “Luigi Vanvitelli”, Napoli, Italy; ^3^Gastroenterologia, Dipartimento di Internistica Clinica e Sperimentale “F. Magrassi”, Università degli Studi della Campania “Luigi Vanvitelli”, Napoli, Italy

**This article has been corrected:** In Figure 3B, the caspase 3 blot in the SW-48 column is an accidental duplicate of the caspase 3 blot in the HC-15 column. The corrected Figure 3B, obtained using the original data, is shown below. The authors declare that these corrections do not change the results or conclusions of this paper.

Original article: Oncotarget. 2017; 8:68305–68316. 68305-68316. https://doi.org/10.18632/oncotarget.20054

**Figure 3:**
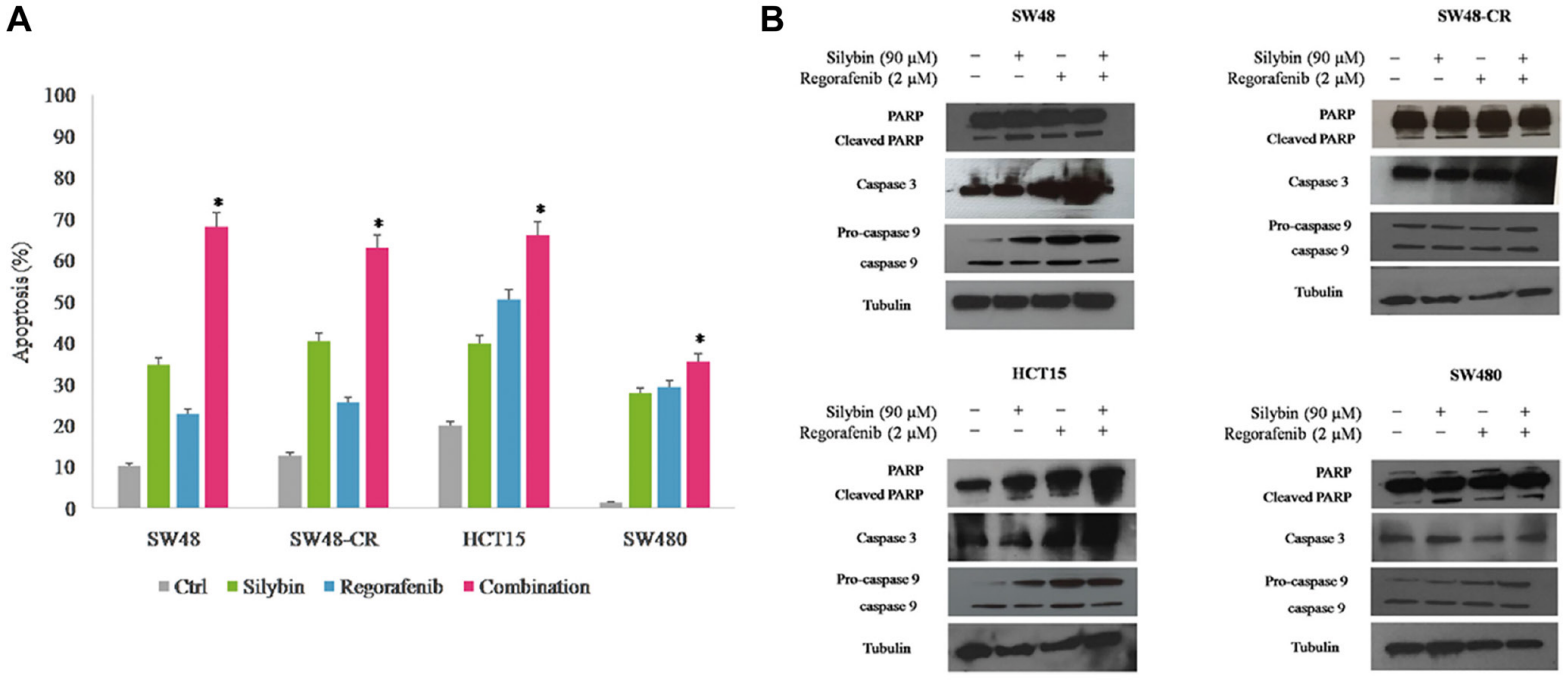
Effects of regorafenib in combination with silybin on induction of apoptosis in SW48, SW48-CR, HCT15 and SW480 colon cancer cells. (**A**) Apoptosis was evaluated with Annexin-V-FITC staining and 7-Amino-Actinomycin D (7-AAD) detection assays using flow cytometry in SW48, SW48-CR, HCT15 and SW480 cancer cells after 24 hours of incubation with silybin (90 μM) or regorafenib (2 μM) and their combination. Histogram of data expressed as percentage of apoptotic cells.^*^ *p* < 0.05 compared to single treatment. (**B**) Colon cancer cells were treated with silybin (90 μM) or regorafenib (2 μM) and their combination for 24 hours. Expression of PARP, caspase 3 and 9 were evaluated by immunoblotting as described in Materials and Methods. α-Tubulin was used as the loading control.

