# Supplementary material for: Regorafenib in combination with silybin as a novel potential strategy for the treatment of metastatic colorectal cancer
Source: Oncotarget. 2017 Aug 7;8(40):68305–16. doi: 10.18632/oncotarget.20054 (PMC5620258; doi:10.18632/oncotarget.20054)
Supplement: Supplementary file 1 [file oncotarget-08-68305-s001.pdf]

Regorafenib in combination with silybin as a novel potential strategy for the treatment of metastatic colorectal cancer

SUPPLEMENTARY MATERIALS

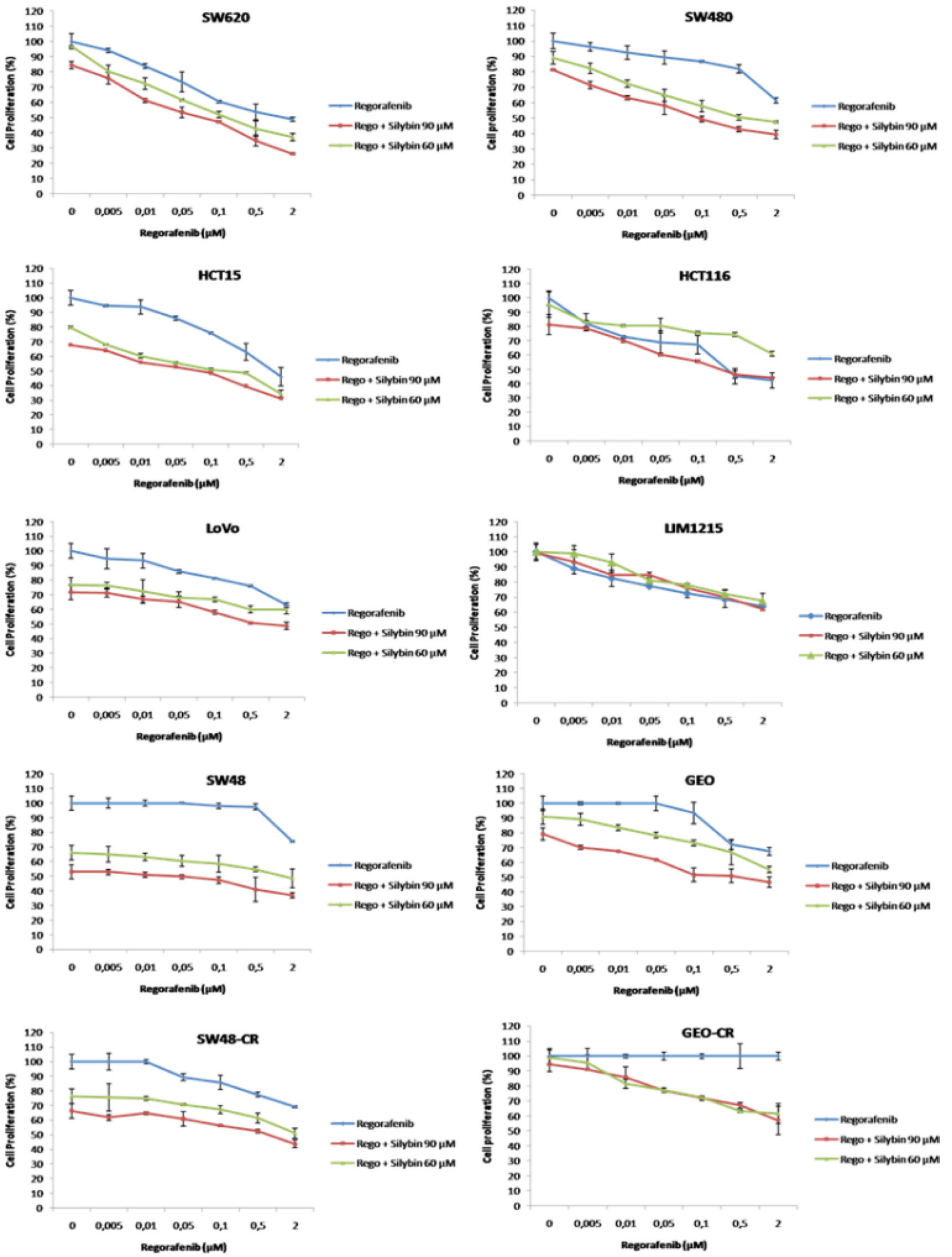

Supplementary Figure 1: Effects of regorafenib in combination with silybin on cell proliferation in a panel of human colorectal cancer cell lines. Human colon cancer cells were treated with two doses of silybin (60 or 90 µM) with increasing concentrations of regorafenib (range, 0.005–2 µM) for 96 hours and evaluated for cell proliferation by MTT assay, as described in Material and Methods. Results represent the median of three separate experiments, each performed in duplicate.

Supplementary Table 1: Patients characteristics

Patients characteristics		Regorafenib + Sylibin *N* = 22	%-(range)
Age (years)		65 years (36–81)	
Sex	Male	11	50
	Female	11	50
Race	Caucasian	22	100
ECOG performance status	0	14	64
	1	5	23
	2	3	14
Primary site of disease	Right colon	4	18
	Left colon	18	82
RAS mutation	Yes	16	73
	No	6	27
Histology	Adenocarcinoma	22	100
Number of previous systemic anticancer therapies (from diagnosis of metastatic disease)	1	1	5
	2	10	45
	3	7	32
	4	4	18
	≥ 5	0	0
Number of metastatic sites	1	3	14
	2	7	32
	≥ 3	10	45
Time from diagnosis of metastatic disease	Median (months)	30.5	(9.0–53.9)
	< 18 months	5	23
	> 18 months	17	77

Supplementary Table 2: Toxicities and dose modification

**Toxicities and Dose modification**		
Starting dose	Patients *N* = 22	
160 mg	18	Basal Hyperbilirubinemia and Hypertransaminasemia (> 3 UNL)
120 mg*	2	Basal Hyperbilirubinemia and Hypertransaminasemia (> 3 UNL) and low ECOG PS
80 mg*	2	
Number of patients with dose reductions	*N* = 18	
Dose reduced from the 1st to 3rd cycle	16	Hyperbilirubinemia and Hypertransaminasemia (85%) HFSR (15%)
Dose reduced after the 3rd cycle	2	HSFR and Fatigue (100%)
Pts requiring 1 dose level reduction: 120 mg Toxicities:	*N* = 6	
Pts requiring 2 dose levels reduction: 80 mg Toxicities:	*N* = 12	
Drug discontinuations due to AEs	*N* = 0	

*no further dose reductions were required after treatment initiation.

Abbreviations: ECOG = Eastern Cooperative Oncology Group; HSFR = hand and foot skin reaction; Pts = patients; PS = performance status.
